# Gene Expression Signature in Peripheral Blood Detects Thoracic Aortic Aneurysm

**DOI:** 10.1371/journal.pone.0001050

**Published:** 2007-10-17

**Authors:** Yulei Wang, Catalin C. Barbacioru, Dov Shiffman, Sriram Balasubramanian, Olga Iakoubova, Maryann Tranquilli, Gonzalo Albornoz, Julie Blake, Necip N. Mehmet, Dewi Ngadimo, Karen Poulter, Frances Chan, Raymond R. Samaha, John A. Elefteriades

**Affiliations:** 1 Applied Biosystems, Foster City, California, United States of America; 2 Celera, Alameda, California, United States of America; 3 Celera Genomics, South San Francisco, California, United States of America; 4 Section of Cardiothoracic Surgery, Yale University School of Medicine, New Haven, Connecticut, United States of America; Stanford University, United States of America

## Abstract

**Background:**

Thoracic aortic aneurysm (TAA) is usually asymptomatic and associated with high mortality. Adverse clinical outcome of TAA is preventable by elective surgical repair; however, identifying at-risk individuals is difficult. We hypothesized that gene expression patterns in peripheral blood cells may correlate with TAA disease status. Our goal was to identify a distinct gene expression signature in peripheral blood that may identify individuals at risk for TAA.

**Methods and Findings:**

Whole genome gene expression profiles from 94 peripheral blood samples (collected from 58 individuals with TAA and 36 controls) were analyzed. Significance Analysis of Microarray (SAM) identified potential signature genes characterizing TAA vs. normal, ascending vs. descending TAA, and sporadic vs. familial TAA. Using a training set containing 36 TAA patients and 25 controls, a 41-gene classification model was constructed for detecting TAA status and an overall accuracy of 78±6% was achieved. Testing this classifier on an independent validation set containing 22 TAA samples and 11 controls yielded an overall classification accuracy of 78%. These 41 classifier genes were further validated by TaqMan® real-time PCR assays. Classification based on the TaqMan® data replicated the microarray results and achieved 80% classification accuracy on the testing set.

**Conclusions:**

This study identified informative gene expression signatures in peripheral blood cells that can characterize TAA status and subtypes of TAA. Moreover, a 41-gene classifier based on expression signature can identify TAA patients with high accuracy. The transcriptional programs in peripheral blood leading to the identification of these markers also provide insights into the mechanism of development of aortic aneurysms and highlight potential targets for therapeutic intervention. The classifier genes identified in this study, and validated by TaqMan® real-time PCR, define a set of promising potential diagnostic markers, setting the stage for a blood-based gene expression test to facilitate early detection of TAA.

## Introduction

Thoracic aortic aneurysm (TAA), without surgical treatment, is a lethal disease [1], [2]; yet, with elective surgical treatment, a near-normal prognosis is restored. Thus, in aneurysm disease, early diagnosis is the key to survival via timely elective surgery. Because TAA is almost invariably asymptomatic until rupture or dissection occurs, detection methods must be applied to asymptomatic individuals. Physical examinations generally do not detect TAA, thus imaging technologies such as echocardiography (ECHO), computerized tomography (CT), or magnetic resonance imaging (MRI) are used for diagnosis of this disease. Although radiographic screening is extremely valuable, many patients who have an increased genetic risk of developing aneurysms later in life may not have recognizable enlargement of the aorta at the time of screening, even with state-of-the-art imaging technologies. Thus a standardized blood-based test capable of detecting individuals at risk for aneurysm disease would represent a major advance in clinical care.

Circulating leukocytes serve as a vigilant and comprehensive surveillance system that patrols the body for signs of infection, inflammation, and structural abnormalities. Therefore, a disease-specific gene expression signature in peripheral blood cells (PBCs) could constitute potential disease markers as well as genes involved in pathogenesis of the disease. PBCs have been used to identify gene expression signatures for autoimmune diseases such as systemic lupus erythematosus (SLE) [3], [4], rheumatoid arthritis (RA) [5], and multiple sclerosis (MS) [3], [6], [7]. These signatures genes have been also shown to be useful in identifying pathways relevant to disease[8] and in predicting response to therapy [9]. Recent studies have also provided evidence that blood cell gene expression profiling might have utility in the detection of complex cardiovascular diseases such as atherosclerosis [10], coronary artery disease [11], [12], and arterial hypertension [13].

Due to the difficulty in obtaining diseased aortic tissue and functional complexity of the system, the mechanisms responsible for the formation of TAA remain elusive; however, the importance of genetic predisposition [14]–[17], inflammation [18]–[21], and adaptive cellular immune responses [22]–[24] in the development of aneurysm disease have been well appreciated. In addition, circulating blood cells are in close contact with the diseased aortic tissue and, therefore, may well serve as reporters for potential disease markers for TAA. For these reasons, it seems likely that gene expression patterns in peripheral blood cells may reflect TAA disease status. In this study, we compared gene expression profiles in peripheral blood cells of individuals with asymptomatic TAA to those of carefully-chosen controls and asked if we can identify a gene expression based TAA-status classifier that can distinguish between individuals with asymptomatic TAA and those without disease.

## Methods

The study was approved by the Yale University Human Investigation Committee (Protocol #12617). All patients gave advance written approval for inclusion in the study.

### Blood samples collection

Peripheral blood was harvested from 58 TAA patients and 36 spousal controls using PAXgene™ tubes (Qiagen, Valencia, CA). All patients (39 male, 19 female) harbored known thoracic aortic aneurysms, based on radiographic images (ECHO, CT, or MRI) and/or operative findings. Since this study focuses on thoracic aortic aneurysms without known underlying disorders, patients with Marfan syndrome were specifically excluded. Spousal controls were chosen because of the similarities in age, ethnicity, geography, and diet that usually characterize husband and wife. Complete blood counts of all blood samples were carried out at the Clinical Laboratory of Yale-New Haven Hospital.

### RNA preparation

The PAXgene™ tubes were frozen at the collection site and shipped on dry ice. After thawing at room temperature for at least 2 hours, total RNA was extracted from the approximately 2.5 ml of peripheral blood in each tube following the manufacturer's recommended protocol (Preanalytix Blood RNA Kit Handbook, Qiagen). The quality and integrity of the total RNA was evaluated on the 2100 Bioanalyzer (Agilent Technologies) and the concentration was measured using a NanoDrop spectrophotometer (NanoDrop Technologies). Globin reduction step was not performed based on the observation that the typical detection rates are similar (55–60% with threshold of S/N>3) between RNA samples directly purified from PAXgene tubes and RNA samples purified from peripheral blood mononuclear cells (PBMC) (data not shown).

#### Applied Biosystems Expression Array analysis

The Applied Biosystems Human Genome Survey Microarray v2.0 (P/N 4337467) contains 33,096 60-mer oligonucleotide probes representing 29,098 individual human genes. cRNA labeling, hybridization and detection were carried out as described previously [25]. For inter-array normalization, we applied Quantile normalization across all microarrays to achieve the same distribution of signal intensities for each array.

#### SAM

Significance analysis of microarrays (SAM; available at http://www-stat.stanford.edu/tibs/SAM/) [26] was used to determine potential signature genes that could distinguish TAA from control samples, or distinguish ascending TAA from descending TAA samples.

#### Hierarchical clustering analysis

Average-linkage hierarchical clustering analysis using centered correlation analysis and visualization was performed using the CLUSTER and TREEVIEW programs (software available at http://genome-www5.stanford.edu/resources/restech.shtml).

#### PANTHER™ Protein Classification System analysis

PANTHER™ (Protein ANalysis THrough Evolutionary Relationships) Protein Classification System (Applied Biosystems, Foster City, CA https://panther.appliedbiosystems.com) classifies proteins in families/sub-families, molecular functions, biological processes and biological pathways [27]. Molecular functions, biological processes and biological pathways over-represented by “signature” genes of the TAA were identified and the statistical significance of the over-representation was quantified by a random overlapping *p* value using the binomial test with all the genes represented by the Applied Biosystems Human Genome Survey Microarray as the reference list [28]. Bonferroni correction for multiple testing was also applied to *p* values used for determining significance in molecular function and biological process.

### Construction and validation of classification models for TAA detection

A 61-sample training set containing 36 TAA patients (24 males and 12 females) and 25 controls (7 males, 18 females) were used to select classifier genes and construct a classification model. Genes were first selected based on the criteria that their expression levels are above the detection threshold (Signal to Noise>3) in≥50% of samples in either TAA or control group. The resulting 16,656 genes from the filtering were then subjected to further gene selection. The classification power for each gene was evaluated using the bootstrap re-sampling method [29] coupled with two-tailed t-statistics. Specifically, during each bootstrap re-sampling process, equal numbers (n = 25) of TAA and control samples were partitioned (repetition allowed) to form a new data set. A two-tailed t-statistic was applied to the new data set and the top 500 genes with the most significant *p*-value were selected. This bootstrap re-sampling process was repeated for 500 times and a total of five hundred 500-gene lists were generated. Genes were then ranked based on their frequency in the 500 500-gene lists and genes with frequency>50% and with average ranking>500 were chosen for further analysis (in general about 105–120 genes). Class prediction was performed by using prediction analysis of microarrays (PAM), a statistical package (http://www-stat.stanford.edu/∼tibs/PAM/) that applies nearest shrunken centroid analysis for sample classification [30]. The optimal number of classifier genes was determined using 10-fold cross validation method on the training set. The 61 (training) samples are partitioned into 10 bins, with equal representation of TAA and controls as the initial set of samples. Nine bins are used for learning purposes to generate an ordered gene list (as described above) based on the gene's probability to be ranked in top 500 most discriminative genes. For any set of top 1, 2, 3, …n genes of this ordered list of genes, classification models were built using the 9 (learning) bins and the TAA status of samples belonging to the remaining bin was classified. Using clinical diagnostics as the reference, true positives (TP), true negative (TN), false positive (FP), and false negative (FN) were calculated. The classification performance was evaluated using the following statistics:
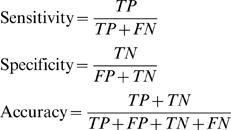



### TaqMan® real-time PCR assay validation

Out of 94 samples analyzed by microarrays, only 82 samples (52 training samples, 30 testing samples) had enough RNA materials to perform TaqMan® real-time PCR assay validation. mRNA expression of 71 genes was measured in each of the 82 samples by real-time PCR using TaqMan® Gene Expression Assays and the 7900HT Fast Real-Time PCR System (Applied Biosystems, Foster City, CA). cDNAs were generated from 30 ng of total RNA using the High Capacity cDNA Reverse Transcription Kit (Applied Biosystems, Foster City, CA). The resulting cDNA was subjected to a 14-cycle PCR amplification followed by real-time PCR reaction using the manufacturer's TaqMan® PreAmp Master Mix Kit Protocol (Applied Biosystems, PN 4366127). Four replicates were run for each gene for each sample in a 384-well format plate. Between the two measured endogenous control genes (PPIA (Alias: cyclophilin A) and MGB2), we chose PPIA for normalization across different genes based on the fact that this gene showed the most relatively constant expression in different breast carcinomas (data not shown). Based on TaqMan assay data, the coefficients of the 41 classifier genes were re-learned from the 52 training samples and used to classify the 30 testing samples using the same method applied to microarray data.

#### GEO accession

The complete data sets of this study including both microarray and TaqMan® assay data can be accessed from GEO with accession number GSE9106. (http://www.ncbi.nlm.nih.gov/projects/geo/)

## Results

### Gene expression signature of TAA in PBCs

PBCs from 58 TAA patients and 36 spousal controls were analyzed in this study. The clinical and demographic characteristics of patients and controls are summarized in Table 1. All subjects of this study are Caucasians. Complete blood cell counts, including white blood cell (WBC), neutrophils, lymphocytes, monocytes, eosinophils and basophils, were determined in all blood samples collected from TAA patients and controls. None of these specific cell counts demonstrated significant association with the TAA disease status based on logistic regression analysis (data not shown).

**Table 1:** Summary of clinical characteristics of 94 TAA patients and controls.

	Training set (n = 61 )		Testing set ( n = 33 )	
	TAA	Control	TAA	Control
**Individual**	36	25	22	11
**Male|Female**	24|12	7|18	15|7	5|6
**Median age (range)**	71 (41–88)	63 (44–87)	62 (32–82)	66 (52–86)
**Average size**	5.3 cm	NA	5.0 cm	NA
**Location (Asc|Dsc|Other)**	31|5|0	NA	16|4|2	NA
**Smoker**	11	4	9	4
**Familial**	7	NA	5	NA

**Figure 1:**
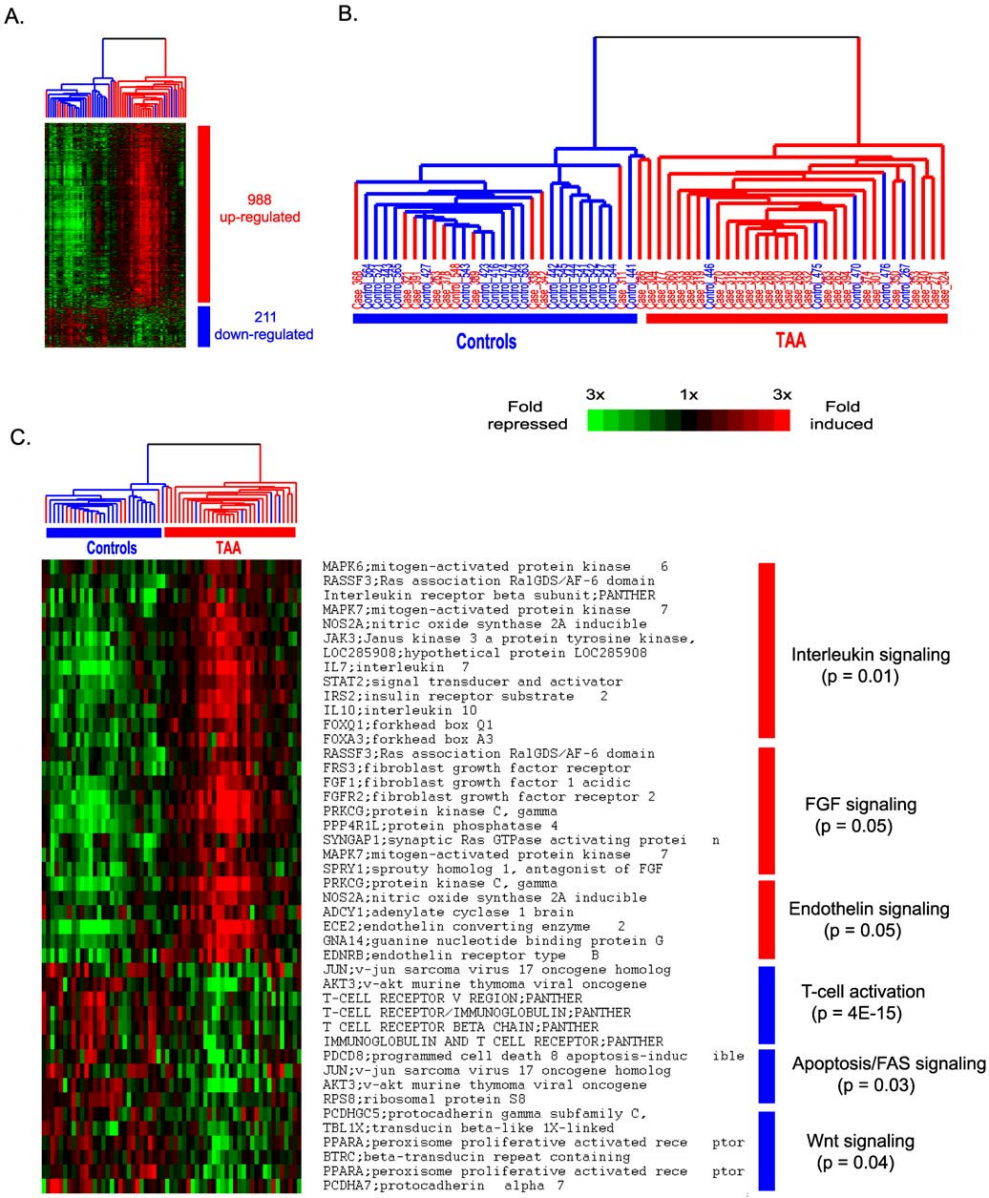
Hierarchical clustering of 61 whole blood samples analyzed by Applied Biosystem Expression Arrays using the 1199 differentially expressed genes determined by SAM analysis. The level of expression of each gene in each sample, relative to the mean level of expression of that gene across all the samples, is represented using a red-black-green color scale as shown in the key (green: below mean; black: equal to mean; red: above mean). (A). Scaled down representation of the entire cluster of the 1199 signature genes and 61 whole blood samples. (B). Experimental dendrogram displaying the clustering of the samples into two main branches: the TAA branch (red) and the control branch (blue) with a few exceptions. (C). Gene expression pattern of representative genes within biological pathways that are statistically significantly over-represented (random overlapping *p*-value<0.05) by the up-regulated (red bars) or the down-regulated (blue bars) signature genes of TAA.

To explore whether we can identify a gene expression signature of TAA disease from peripheral blood samples, gene expression profiles of 61 whole-blood RNA samples (training set) collected from 36 TAA patients and 25 controls were analyzed using the Applied Biosystems Human Genome Survey Microarrays representing 29,098 individual human genes. Using SAM analysis, 1207 genes were differentially expressed between the TAA and control groups based on the following criteria: (1) false discovery rate (FDR)<4% from 300 permutation testing; and (2) average fold change between the TAA patients and controls>1.3-fold. To examine whether the imbalanced gender distribution within the TAA and control groups (Table 1) may confound the identification of TAA signature genes, SAM analysis was performed between the 31 male and 30 female samples within the training set. Twenty-eight genes were identified as gender-specific genes using the same criteria (FDR<4% and>1.3-fold between the two gender groups). Not surprisingly, 21 out of the 28 gender-specific genes were found located on either Y or X chromosome. Eight of the 28 gender specific genes (5 Y-linked and 3-X linked) were also found in the list of 1207 differentially expressed genes and were excluded from further analysis. Figure 1A displays hierarchical clustering diagrams of the 61 training set samples using the remaining 1199 potential TAA signature genes, among which, 988 genes were over-expressed and 211 genes were under-expressed in TAA patients when compared to the control samples (Figure 1A, complete gene list is listed in Table S1). These signature genes generally clustered the TAA patients and the controls into two distinct branches with only a few exceptions (Figure 1B).

PANTHER™ Protein Classification System analysis[27] showed that after correction for multiple hypothesis testing there were statistically significant enrichments of transcripts within the 988 genes that are up-regulated in the TAA patients, displaying interleukin signaling (*p*-value = 0.01), the fibroblast growth factor (FGF) signaling (*p*-value = 0.05), and the endothelin signaling activities (*p*-value = 0.05) (Figure 1C). The over-expressed genes included the Th2-derived cytokine IL-10 and other genes involved in the downstream JAK/STAT signaling cascade, including tyrosine kinase JAK3, map kinases (MAPK6, MAPK7) and signal transducer and activator of transcription STAT2, suggesting a coordinated Th2-activated immune response associated with the circulating cells of the TAA patients. On the other hand, statistically significant association of the 211 down-regulated genes in TAA patients was observed with pathways such as T-cell activation (*p*-value = 4E-15), Fas-mediated apoptosis (*p*-value = 0.03), and Wnt signaling (*p*-value = 0.04) (Figure 1C).

### Construction of classification model for detection of TAA

**Figure 2:**
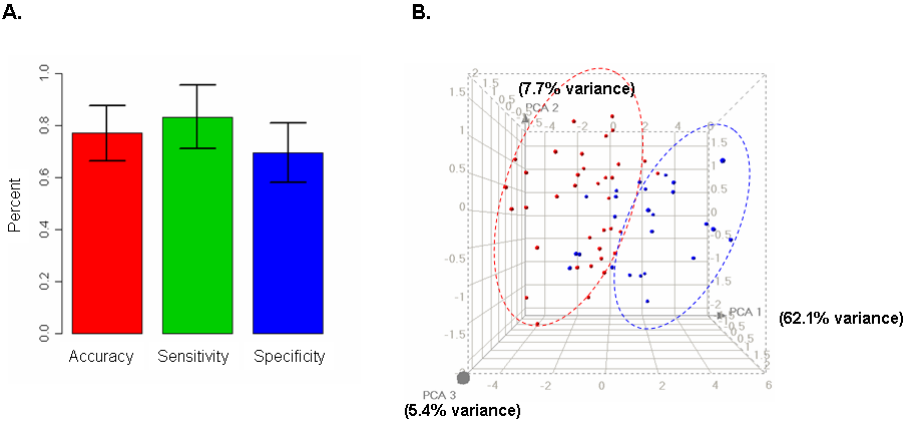
A set of 41 classifier genes were identified via 10-fold cross-validation on the 61-sample training set. (A). Classification accuracy, sensitivity and specificity of the 41 classifier genes, error bar represents±1 standard deviation among 100 times of independent 10-fold cross-validation process; (B). 3D Plots of the first three principal components based on PCA analysis. The segregation between TAA and control samples is evident with only a few exceptions.

Our goal was to identify a distinct gene expression signature in peripheral blood that may allow development of noninvasive screening tests to identify individuals at risk for TAA disease. Due to the high inter-individual variability, we applied a bootstrapping strategy [29] that takes into account of the complex relationship between genes, as well as the variability between samples. Such a strategy minimizes the impact of extreme outlier samples and allows confidence estimations for classifier gene selection. Classifier gene selection was done by evaluating each gene using a bootstrap re-sampling method coupled with two-tailed t-statistics (described in the Methods). The discriminative power of each gene was ranked during each bootstrap re-sampling process and this bootstrap re-sampling process was repeated for 500 times. The most discriminative genes (∼100) were identified using the following criteria: (1) with>50% frequency in appearing in the top 500 ranks among the 500 bootstrap re-sampling; (2) with average rank<500. To determine an optimal number of classifier genes, we started from the selected 100 genes and removed one gene at a time and estimated the corresponding classification accuracy using a 10-fold cross-validation on the training set [30]. A 41-gene classifier was identified based on its consistency in classification accuracy (Figure S1). 10-fold cross-validation of the 41-gene model on the training set yielded an overall classification accuracy (average 78±6%), sensitivity (average 81±6%) and specificity (average 75±6%)(Figure 2A). Principal component analysis using the 41 classifier genes can segregate TAA samples from control samples in three dimensional spaces with only a few exceptions (Figure 2B). The list of the 41 classifier genes is presented in Table 2.

**Table 2:** List of the 41 classifier genes that distinguish TAA from normal individuals.

Array Probe ID	Primary Gene ID	Gene Symbol	Frequency in top 500 ranks among 500 bootstrap resampling	Expression in TAA
124958	56143	PCDHA5	0.82	Up
227481	374897	SBSN	0.82	Up
182618	hCG1773879.2		0.80	Up
219146	348932	SLC6A18	0.77	Up
211834	hCG1820398.1		0.77	Up
214799	219968	OR5B21	0.77	Up
619119	388751	LOC388751	0.76	Up
196612	22875	ENPP4	0.72	Up
213445	8809	IL18R1	0.72	Up
208499	283349	RASSF3	0.71	Up
410937	387695	C10orf99	0.71	Up
163657	8831	SYNGAP1	0.69	Up
183110	hCG2028451.1		0.65	Up
137330	hCG1820722.2		0.64	Up
141979	5998	RGS3	0.64	Up
115931	64100	ELSPBP1	0.63	Up
214599	29957	SLC25A24	0.60	Up
192269	6944	VPS72	0.96	Down
117989	3615	IMPDH2	0.93	Down
139001	3033	HADHSC	0.92	Down
206569	6631	SNRPC	0.88	Down
156536	5245	PHB	0.88	Down
125597	79001	VKORC1	0.87	Down
179149	3163	HMOX2	0.82	Down
179328	8607	RUVBL1	0.82	Down
162516	231	AKR1B1	0.78	Down
201766	6184	RPN1	0.77	Down
115653	8721	EDF1	0.77	Down
185037	1019	CDK4	0.77	Down
178575	128240	APOA1BP	0.75	Down
154562	25764	HYPK	0.71	Down
175040	516	ATP5G1	0.68	Down
187195	29101	SSU72	0.67	Down
119963	10001	MED6	0.66	Down
232086	hCG2042278		0.65	Down
173844	4282	MIF	0.65	Down
180941	hCG1793363.2		0.62	Down
112601	79228	WDR58	0.62	Down
224173	83858	ATAD3B	0.61	Down
204089	51070	NOSIP	0.61	Down
178334	11164	NUDT5	0.59	Down

**Figure 3:**
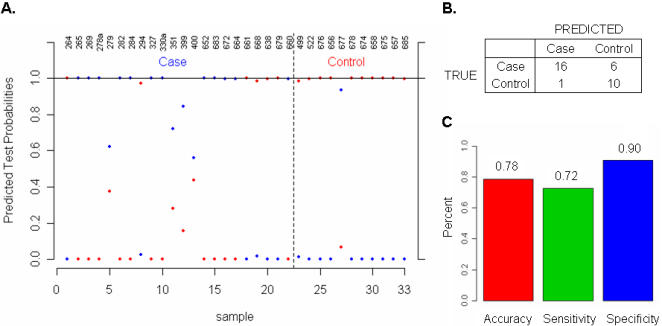
Validation of the 41-gene classification model using microarray analysis on an independent set of testing samples. (A). Classification probabilities for each testing sample: TAA (case) vs. normal (control); (B). Contingency table summarizes the predicted and actual class membership for the testing set; (C). Classification accuracy, sensitivity and specificity.

As an independent measure of the validity of the 41-gene classification model, we tested an independent cohort consisting of 33 peripheral blood samples (22 TAA and 11 controls), more than half of which were collected at least one year after the completion of the first set. RNAs extracted from these samples were analyzed using Applied Biosystems Human Genome Arrays using the same protocols as the training set, but by different operators and instruments. The classification accuracy, sensitivity, and specificity for the testing set were 78%, 72%, and 90% respectively (Figure 3), consistent with the results estimated by the 10-fold cross validation on the training set (Figure 2A).

### TaqMan® real-time PCR assay validation

**Figure 4:**
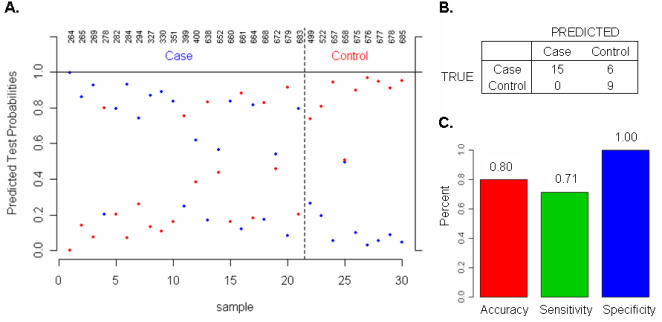
Validation of the 41 classifier genes using TaqMan real-time PCR assays. The expression profile of the 41 classifier genes was measured in each of the 82 samples by real-time PCR using TaqMan® Gene Expression Assays. Based on TaqMan assay data, the coefficient of the 41 classifier genes were re-learned from the 52 training samples and used to classify the status of 30 testing samples using the same method applied to microarray data. (A). Classification probabilities for each testing sample: TAA (case) vs. normal (control); (B). Contingency table summarizes the predicted and actual class membership for the testing set; (C). Classification accuracy, sensitivity and specificity.

To further validate the TAA-status classifier genes, we determined expression levels of the 41 TAA-status classifier genes using the more precise real-time PCR methodology on 82 samples for which enough RNA was available (50 samples from training set and 32 samples from test set). Similar classification performance was achieved using real-time PCR data compared to that of microarray data: the classification accuracy, sensitivity and specificity for the testing set based on TaqMan® assay data were 80%, 71%, and 100% respectively (Figure 4).

### Molecular profiles characterizing subtypes of TAA

One of the main clinical characteristics of TAA is the location of the aneurysm (i.e. in ascending vs. descending aorta). Aneurysms in these two locations tend to represent very different clinical phenomena [31]. The location of an aneurysm is distinctly connected with the embryology, pathogenesis, clinical course, and treatment of a thoracic aneurysm.

**Figure 5:**
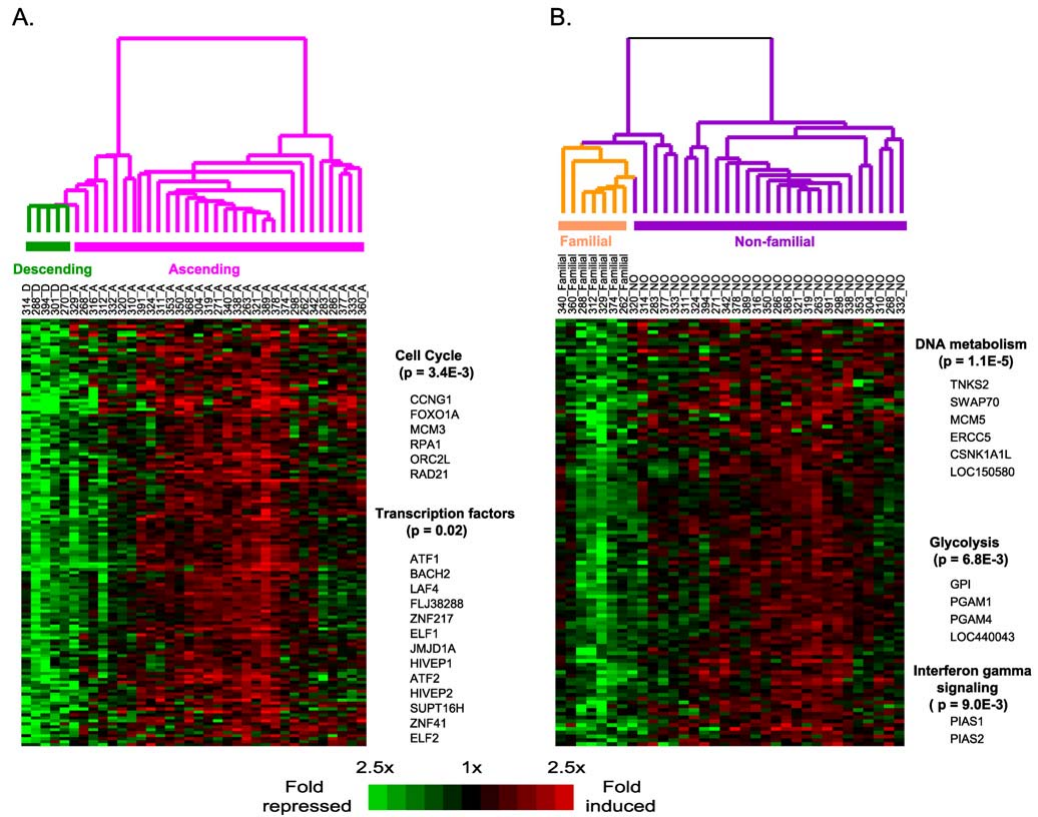
Two-dimensional cluster diagrams. (A).144 signature genes characterizing the ascending and descending TAA subtypes; (B). 113 signature genes characterizing the TAA with or without family history. Representative genes associated with over-represented molecular functions/biological processes/pathways are listed.

To explore whether we can identify signature expression profiles characterizing location-specific TAA, SAM analysis was performed on 36 TAA samples within the training set (31 ascending vs. 5 descending). One hundred and forty-four genes were significantly over-expressed in the ascending TAA samples (FDR<2%, FC>1.3) (Table S2). Hierarchical clustering analysis of these 144 genes grouped the descending and ascending TAA samples into two distinct and separate clusters (Figure 5A). PANTHER™ Protein Classification System analysis revealed that genes involved in cell cycle regulation (i.e. RAD21, ORC2, LRPA1, MCM3, FOXO1A, and CCNG1) are significantly over-represented in the set of 144 genes that are differentially expressed in samples from patients with ascending TAA (*p*-value = 3.4E-3). This set also contained thirteen transcription factors (*p*-value = 0.02, Figure 5A), including ELF1 and ELF2, two transcription factors involved in the platelet derived growth factor (PDGF) signaling pathway , which plays a critical role in cellular proliferation and development.

Familial aggregation studies indicate that up to 20% of patients with TAA (who do not have Marfan syndrome) have a first-degree relative with the disease[1], [31]–[33]. To investigate potential gene expression signatures characterizing familial and sporadic TAA, we performed SAM analysis on 7 TAA patients with a family history of the disease and 27 TAA patients without a family history of the disease (sporadic), all from within the training set. One hundred and thirteen genes were identified as significantly up-regulated in patients with sporadic TAA (average FC>1.3 and FDR<4%) and hierarchical clustering based on these genes clustered the familial and sporadic TAA patients into distinct branches (Figure 5B) (Table S3). PANTHER™ Protein Classification System analysis revealed that this set of genes up-regulated in patients with sporadic TAA was statistically enriched for genes involved in various aspects of DNA metabolism (*p*-value = 1.1E-6), including DNA replication (LOC150580, MCM5, TNKS2), DNA repair (ERCC5, CSNK1A1L) and DNA recombination (SWAP70). Several genes involved in glycolysis (PGAM1 and GPI) and interferon signaling (PIAS1,PIAS2) were also significantly up-regulated within the sporadic TAA peripheral blood. Another interesting gene specifically down-regulated in familial TAA is the angiogenic factor AGGF1. This gene, located at chromosome 5q13.3, is within the previously mapped 5q13–14 locus associated with familial TAA [14], [34].

Twenty eight signature genes characterizing sub-types of TAA were also validated by TaqMan real time PCR assays. Even though the average fold changes for most of these signature genes are relatively small (mostly<2-fold), these were reproducible, as shown by 89% concordance between the TaqMan assay data and the microarray data (Table S4).

Finally, SAM analysis was also performed to identify signature genes correlated with age, aneurysm size and smoking status of TAA patients; however, no genes were found to correlate significantly with these factors (data not shown).

## Discussion

It has become increasingly evident that the immune system plays a pivotal role in the development of aortic aneurysms [18]–[24]. We thus hypothesized that gene expression patterns in peripheral blood cells may reflect TAA disease status. The specific aim of this study was to obtain a distinct molecular signature that can signal the presence of TAA and potentially identify at-risk patients for further clinical and radiological testing. Through a whole-genome gene expression profiling analysis in a relatively large number of thoracic aneurysm patients and controls, we have identified a 41-gene signature in peripheral blood cells that distinguishes TAA patients from controls. This set of 41 classifier genes, derived from microarray analysis and further validated by TaqMan® real-time PCR assays, detects TAA disease with an overall classification accuracy of 78–80%, which is a promising level for potential clinical applications.

Moreover, this investigation also provided a comprehensive analysis of the gene expression programs that characterize the peripheral blood cells of TAA patients, which may reflect activities of the immune cells at the site of the disease. It is encouraging, therefore, that many of the biological pathways (and genes) identified in this study, such as interleukin signaling, T-cell activation and apoptosis, have been associated with development of aneurysm diseases [18], [23], [24], [35]. For example, the interleukin signaling pathway has been particularly well studied in the aneurismal context [18]–[21], [36], [37]. Both pro- and anti-inflammatory cytokines have been postulated to play vital roles in development, rupture and repair of aneurysms [36], [37]. These include interferon-gamma (IFN-gamma), interleukin-6 (IL-6), and interleukin-10 (IL-10). It is interesting that the major pro-inflammatory cytokines IL-6 and IFN-gamma did not appear among the significantly differentially expressed genes in our study. In contrast, our results revealed an over-expression of IL-10 in the peripheral blood cells of TAA patients, as well as a coordinated up-regulation of several components of the downstream signaling pathways, namely various members of the JAK/STAT/MAPK families. IL-10 is, like most interleukins, a pleiotropic cytokine, but is somewhat unusual in that its actions are primarily anti-inflammatory. It is secreted mainly by monocytes and the Th2-subset of CD4+ T-cells and is capable of polarizing the inflammatory response towards a Th2 type; this latter property might play a role in aneurysm since it has been reported that a Th2-type inflammation and blockade of the Th1 cytokine IFN-gamma induce aneurysms in allografted aorta in mice [38]. Over-expression of IL-10 has also been noted in aortic tissue from abdominal aortic aneurysm (AAA) patients compared to normals or carotid atheroma patients [24], [39]. Thus over-expression of IL-10 could, in this scenario, inhibit the Th1 response and increase the Th2 contribution, leading to the development of aneurysm. However, there is a large body of evidence to show that a Th1-directed inflammation and IFN-gamma release activate macrophages to secrete MMPs and accelerate the onset of tissue degeneration leading to aneurysm [18], [40]. And in fact, IL-10 has indeed been reported to limit the cytotoxic potential of macrophages and to reduce the expression of pro-inflammatory mediators such as cytokines or matrix metalloproteinases (MMPs) [41], [42]. It is also capable of driving down the Th1 response, and directly inhibiting IFN-gamma; thus it is likely that IL-10 over-expression is in fact a beneficial immune response to counteract the MMP production and limit injury. This view is in agreement with the reported increase in IL-10 in pre-operative AAA [36] and observations of increased IL-10 immediately following surgery; in fact, a Phase I clinical trial has been initiated to examine the benefit of IL-10 administration following TAA repair surgery [43]. This view of Th1-directed aneurysm and later increase of IL-10 and Th2-directed response is in line with the known anti-inflammatory properties of IL-10, as has been suggested [44]. Another example of this protective immune response is the down-regulation of the T-cell activation pathway observed in this study, primarily occurring by down-regulation of subunits of the T-cell receptor and its immediate downstream signaling components. Again, this could be another late mechanism for modulating the inflammatory stimulus and limiting subsequent tissue damage.

The observed down-regulation of members of the Fas/apoptosis pathway such as AKT3 in TAA could also be related to the reported apoptosis of vascular smooth muscle cells (VSMC), which might otherwise repair the tissue damage described above [35]. AKT3 is known to regulate Fas sensitivity and promote cell survival [45]. Down-regulation of Akt signaling may induce Fas ligand expression and promote Fas-mediated apoptosis in VSMC [46]. Another interesting finding of this study is the endothelin pathway, including endothelin-converting enzyme 2 (ECE-2) was up-regulated in TAA. Endothelins are well known to be potent vasoconstrictors that play a major role in vasospasms following cerebral aneurysms [47], and inhibitors of endothelin-converting enzymes are in development for prevention and treatment of these conditions. The potential involvement of endothelin-converting enzymes in the pathogenesis of thoracic aneurysms has not received much attention, and may merit further study.

While the statistically significant pathways emerging from the transcriptional analysis may provide mechanistic insights, many of the individual genes in these pathways are not among those in the 41-gene marker set. As mentioned earlier, this may be because many of these pathway genes are commonly involved in many other processes in addition to aneurysms and may not be sufficiently discriminatory for the particular function of distinguishing TAA PBC from normal. However, some of the genes in the 41-gene classifier list can be related mechanistically to aneurysm–for example, macrophage migration inhibition factor (MIF), is released by immune cells, and is a potent stimulator of Th1 cells, inducing the release of pro-inflammatory cytokines including IFN-gamma. High levels of MIF have been correlated with AAA, which is also common in TAA patients following surgery [48], [49]. The observed down-regulation of MIF in PBC from this study could therefore be a beneficial response.

This investigation also identified distinct gene expression profiles for subtypes of TAA, including ascending vs. descending TAA and familial vs. sporadic TAA. It is worth noting that these profiles were derived from relatively small numbers of samples, therefore they should be considered as preliminary observations on expression pattern of these subtypes of TAA. The classification gene set needs to be further established and cross-validated in studies with larger sample sizes. On the other hand, these results provide further evidence supporting the current understanding that ascending and descending thoracic aortic aneurysms may represent two very different diseases with widely differing, pathophysiology, and clinical manifestations [50] and, therefore, may need different therapeutic strategies. It is also consistent with the increasing recognition that TAA disease is indeed transmitted in an inherited fashion in at least one-fifth of patients [31] and opens many interesting avenues to explore in future studies. One particular interesting finding of this study is the angiogenic factor AGGF1 gene, which was identified as one of the potential signature genes distinguishing familial vs. sporadic TAA. AGGF1 is located at chromosome 5q13.3, which is mapped within the previously mapped 5q13–14 locus that has been shown to be associated with familial TAA [14], [34]. Therefore, AGGF1 should be evaluated as a candidate gene for TAA. Thus, our gene expression profiles of PBC may provide additional information to genetic studies of TAA and help to identify potential disease-associated genes for familial TAA. Finally, our identification of a specific signature for sporadic TAA also holds special promise in general screening of TAA disease, because traditional radiographic imaging methods or genetic tests that are potentially applicable to known affected families cannot be applied to sporadic cases due to the low cost-effectiveness.

There are potential limitations of this study that should be considered. First, the choice of spouses as controls for this study may be sub-optimal; however, the advantage of using spousal controls over randomly chosen normal individuals is that they share many demographic and environmental factors such as age, geography, and diet. Secondly, while the TAA signature genes identified in PBC may provide clues to pathogenic processes of the disease, any biological pathways underlying these signature genes need to be further evaluated in mechanistic studies derived from diseased aortic tissues. It must be reiterated that the strength of this study lies primarily in the identification of disease markers in peripheral blood cells, which is a preferred choice for the purpose of early diagnosis of TAA. Third, it remains to be determined whether the TAA signature can be applied to detect abdominal aortic aneurysms (AAA), especially since it has been shown that patients with descending TAA were likely to have kindred with AAA [33]. Preliminary analysis showed no significant overlapping between the TAA signature genes identified in this study and those reported for other vascular diseases such as atherosclerosis [10], coronary artery disease[11], [12] and arterial hypertension [13], suggesting that this signature is specific to TAA rather than a general signature for vascular disease or inflammation. Finally, although we believe the TAA classifier genes identified in this study define potential diagnostic markers for TAA, we want to emphasize that further study needs to be done before clinical application, including independent validation (retrospective and prospective) with larger numbers of subjects and determining the positive prediction value (PPV) and negative prediction value (NPV) in the context of a clinical diagnostic test.

In conclusion, we have identified informative gene expression signatures in peripheral blood cells that can distinguish TAA status and subtypes of TAA. From these data, we have identified a signature set of 41 biomarker genes which can identify asymptomatic TAA patients with high accuracy. This can potentially lead to a diagnostic “point-of-care” assay, for identification of asymptomatic TAA disease with a simple blood-based test. The transcriptional programs in peripheral blood leading to the identification of these markers also provide insights into the mechanism of development of aortic aneurysms, and highlight potential targets for therapeutic intervention. We are hopeful that the findings of this investigation can lead to better and earlier diagnosis of the aneurysm diathesis, thus protecting humans from this lethal disease.

## Supporting Information

Figure S1 Determination of the optimal set of classifier genes using 10-fold cross-validation on the training set (see detailed description in Methods). Classification accuracy using different number of classifier genes was illustrated; the error bar indicates±1 SD among 100 times of independent 10-fold cross-validation process.
(0.26 MB TIF)

Table S1 1199 significantly differentially expressed genes distinguishing TAA vs. control, identified based on microarray data and SAM analysis (ave. FC>1.3 and FDR<4%)
(0.22 MB XLS)

Table S2 144 candidate signature genes distinguishing ascending vs. descending TAA, identified based on microarray data and SAM analysis (ave. FC>1.3 and FDR<2%)
(0.04 MB XLS)

Table S3 113 candidate signature genes distinguishing familial vs. sporadic TAA, identified based on microarray data and SAM analysis (ave. FC>1.3 and FDR<4%)
(0.03 MB XLS)

Table S4 Validation of signature genes charactering sub-types of TAA using TaqMan® real-time PCR assays. The signature genes were originally identified by microarray using SAM analysis (average fold change>1.3 and FDR<4%)
(0.02 MB DOC)
