# Supplementary figures and images for: Gene Expression Signature in Peripheral Blood Detects Thoracic Aortic Aneurysm
Source: PLoS One. 2007 Oct 17;2(10):e1050. doi: 10.1371/journal.pone.0001050 (PMC2002514; doi:10.1371/journal.pone.0001050)

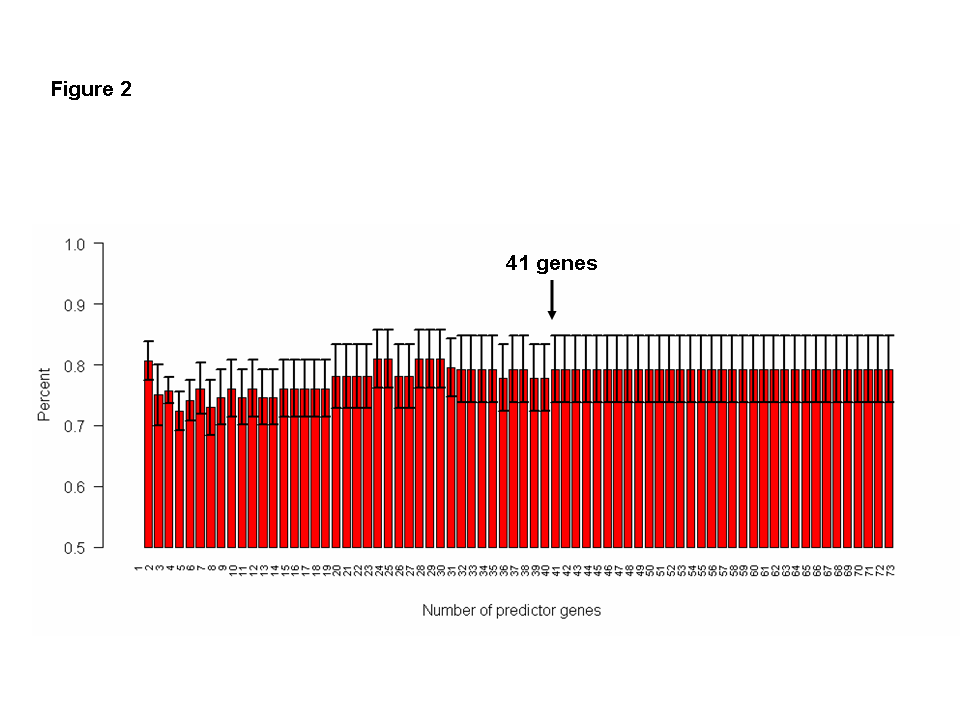

Supplement: Figure S1 — Determination of the optimal set of classifier genes using 10-fold cross-validation on the training set (see detailed description in Methods). Classification accuracy using different number of classifier genes was illustrated; the error bar indicates±1 SD among 100 times of independent 10-fold cross-validation process. (0.26 MB TIF) [file pone.0001050.s001.tif]
