# Supplementary material for: Gene Expression Signature in Peripheral Blood Detects Thoracic Aortic Aneurysm
Source: PLoS One. 2007 Oct 17;2(10):e1050. doi: 10.1371/journal.pone.0001050 (PMC2002514; doi:10.1371/journal.pone.0001050)
Supplement: Table S4 — Validation of signature genes charactering sub-types of TAA using TaqMan® real-time PCR assays. The signature genes were originally identified by microarray using SAM analysis (average fold change>1.3 and FDR<4%) (0.02 MB DOC) [file pone.0001050.s005.doc]

A. Selected signature genes distinguishing ascending vs. descending TAA

B. Selected signature genes distinguishing familial vs. sporadic TAA
